# Effect of Calcareous Material Particle Size via Separate Grinding on the Burnability and Microstructure Development of Portland Cement Clinker

**DOI:** 10.3390/ma19101935

**Published:** 2026-05-08

**Authors:** Xin Du, Ruizhi Zhang, Suping Cui, Minghao Liu, Wenhai Nie, Yali Wang, Xuyue Liu, Hui Liu

**Affiliations:** 1 College of Materials Science & Engineering, Beijing University of Technology, Beijing 100124, China; wangyali1978@bjut.edu.cn (Y.W.); liuhui9516@bjut.edu.cn (H.L.); 2 Tianjin Cement Industry Design & Research Institute Co., Ltd., No. 36 Benxi Road, Tianjin 300131, China; zhangruizhi@sinoma-tianjin.cn (R.Z.); liuminghao@sinoma-tianjin.cn (M.L.); niewenhai@sinoma-tianjin.cn (W.N.); liuxuyuen@sinoma-tj.cn (X.L.)

**Keywords:** cement raw meal, burnability, particle size, *f-CaO*, clinker mineralogy, semi-empirical reaction kinetics

## Abstract

**Highlights:**

- Reveals a crucial calcium particle size threshold (*R*_80__μm_).
- Particles >200 μm exert a stronger influence on *f-CaO* than those >80 μm.
- Multi-scale characterization of clinker mineral composition and microstructure.
- Couples temperature and particle size in a semi-empirical reaction kinetics model.

**Abstract:**

Based on the separate grinding process for raw meals in the cement industry, raw meal samples with different particle size characteristics were prepared by controlling the fineness of calcareous components. The results show that the fineness of the calcareous components has a significant influence on the burnability of the clinker and that a critical threshold exists (80 μm sieve residue (*R*_80μm_) = 15%). When the particle size exceeds this critical value, the particle size effect becomes dominant, leading to a nonlinear and sharp increase in f-CaO content. As the proportion of coarse particles larger than 200 μm increases, the f-CaO content rises markedly, with a greater impact than that of 80 μm particles. Microscopic analysis of the clinker reveals that with coarsening of the calcareous components (increase in *R*_80μm_), alite (C_3_S) content decreases, whereas belite (C_2_S) and f-CaO contents gradually increase and exhibit enrichment. Based on diffusion-controlled kinetics, a semi-empirical reaction kinetics model, $f\text{-}\mathrm{CaO}\;=\;A\cdot \exp\frac{\left(E_{a,0}+k\cdot R_{80\;\mu m}\right)}{\mathrm{RT}}\cdot ({R_{80\mu m})}^{n}$, was developed by introducing the apparent activation energy parameter *E_a_*(*R*_80μm_) as a function of particle size. The model exhibited excellent goodness of fit (*R*^2^ > 0.95), with an intrinsic activation energy *E_a,_*_0_ = 18.7 kJ·mol^−1^ and an incremental coefficient *k* = 0.28 kJ·mol^−1^·%^−1^. Validation experiments yielded a relative error of 4.3%. This model quantifies the coupled effects of temperature and particle size, providing quantitative guidance for balancing grinding energy consumption and sintering energy consumption.

## 1. Introduction

The global cement industry, as a cornerstone of infrastructure development, is simultaneously a significant consumer of energy and a major contributor to anthropogenic CO_2_ emissions, accounting for 6–8% of the global total [1,2,3]. Facing the dual pressures of the energy crisis and climate change, this sector has strategically pivoted to deep decarbonization. A paramount focus within this shift is enhancing energy efficiency in clinker production [4]. The formation of clinker involves three principal stages: (1) decomposition of limestone to produce CaO; (2) reaction between CaO and SiO_2_ to form belite (C_2_S); and (3) further reaction of C_2_S with additional CaO to yield alite (C_3_S). This reaction sequence is influenced by multiple factors, including temperature, particle size of raw materials, and diffusion kinetics. The ease of clinker burning is commonly assessed by the burnability of the raw meal, which directly reflects the thermal energy demand during cement production and, consequently, affects its carbon footprint [5]. Therefore, optimizing the particle size distribution of raw meal offers a cost-effective strategy for process improvement. By reducing sintering temperatures [6], shortening residence time, and lowering final *f-CaO* content, this strategy directly enables significant energy conservation and emission reduction. Crucially, this strategy delivers these benefits without requiring major capital investment in kiln modifications.

However, achieving a uniform and ideal particle size distribution presents fundamental technological challenges. In industrial production, the traditional raw meal preparation process employs combined grinding, with *R*_80μm_ as the control parameter. This method leads to severe heterogeneity in particle size distribution due to the significant differences in hardness and grindability between siliceous (e.g., sandstone) and calcareous (e.g., limestone) raw meals [7]. The siliceous components tend to concentrate in the coarse particle fraction, while the calcareous components are excessively ground into fine particles. This selective breakage reduces the homogeneity of the raw meal. It triggers localized anomalies in the silica modulus during calcination. These anomalies, in turn, significantly inhibit the nucleation and growth of tricalcium silicate (C_3_S). Consequently, this leads to an increase in the *f-CaO* content in the clinker or a rise in heat consumption [8].

To improve the homogeneity and reactivity of raw meal, a separate grinding process has been proposed and applied. The siliceous materials (e.g., sandstone) are ground individually to the specified fineness. The calcareous materials (e.g., limestone, fly ash, iron ore sludge, and coal ash) are first proportioned and then co-ground to the target fineness. Subsequently, all components are thoroughly mixed according to the predetermined ratio. Over the years, research has consistently shown a negative correlation between the overall Blaine specific surface area of raw meal and its *f-CaO* content. This correlation is primarily attributed to enhanced solid-phase diffusion kinetics driven by a higher specific surface area [9,10,11]. Classical studies have primarily focused on the macroscopic relationship between Bogue mineral composition and overall fineness. However, a key limitation arises when materials are prepared by combined grinding: the distinct fineness effects of calcareous (CaCO_3_-rich) and siliceous (SiO_2_-rich) components, along with their calcination synergies, remain obscured [12]. This oversight has led to an insufficiently deep understanding of the reaction pathways, particularly failing to clearly elucidate the independent influence of calcareous material fineness on the burnability of the raw meal and its underlying mechanisms.

Macroscopic empirical rules have been extensively documented. However, multi-scale studies are still lacking. Specifically, research has not yet systematically linked particle-scale traits (especially calcareous component fineness) and calcination regimes to the mineral phase composition (C_3_S, C_2_S, C_3_A, C_4_AF) and the microstructural evolution of clinker [13,14]. This integrated perspective remains absent from current approaches. Consequently, the progression from empirical rules to mechanism-based predictive models is hindered, making targeted process intensification difficult to achieve. This study aims to systematically investigate the independent effect of calcareous material fineness on raw meal burnability. Through combined experimental and multi-scale analyses, the underlying mechanisms were elucidated. The findings are expected to address existing theoretical gaps and provide a scientific foundation for optimizing grinding processes to reduce energy consumption and carbon emissions in cement manufacturing.

## 2. Materials and Methods

### 2.1. Raw Materials and Experimental Design

The chemical compositions of the raw materials, as determined by X-ray fluorescence (XRF) spectrometry, are presented in Table 1. The lime saturation factor (LSF), silica modulus (SM), and alumina modulus (AM) of the designed raw meal were calibrated to 0.91, 2.70, and 1.50, respectively. The values represent a conventional clinker formulation in the Portland cement industry, characterized by good burnability and a homogeneous microstructure. Using it as a baseline to investigate the influence of calcareous particle size ensures the representativeness and comparability of the experimental results. Raw meal proportions are shown in Table 2.

To clarify the effects of calcium component fineness, the study strictly employed separate grinding and mixed-batching operational procedures. The calcareous component (including limestone, fly ash, iron-soil sludge, and coal ash) and siliceous component (sandstone only) were ground to predetermined target fineness levels using a laboratory-scale Φ305 × 305 mm ball mill. The ball mill uses steel balls as the grinding media. The mass of the steel balls is 20.0 kg, and their size distribution is provided in Table 3. During the grinding process, the fineness of the raw meals was measured using a vacuum sieving analyzer equipped with an 80 μm square-hole sieve. The target fineness of the ground materials was achieved by controlling the grinding time.

The final powders were homogenized for 30 min in a laboratory mixer to produce a uniform raw meal. Following the procedure, three distinct sample series were designed and prepared.

L-Series (Varied Calcareous Fineness): The siliceous component fineness *R*_80μm_ was held at 5%, while the calcareous component was ground to achieve a residue on *R*_80μm_ ranging from 1.48% to 37.96%, as shown in Table 4.

LC-Series (Controlled Coarse Calcareous Particles): The siliceous component fineness *R*_80μm_ was held at 5.00%, while the calcareous component was ground to achieve a residue on the *R*_80μm_ ranging from 25.84% to 37.62%, and the *R*_200μm_ around 5%, as shown in Table 5.

### 2.2. Clinker Calcination

The burnability of the prepared raw meals was evaluated using a laboratory-scale simulated clinkering process. Approximately 3.6 g of each homogenized raw meal was uniaxially pressed into Φ13 mm pellets at 10.6 kN. The pellets were subsequently calcined in a high-temperature electric furnace with an air atmosphere. The thermal regime was set as follows: the temperature was raised to 950 °C at a heating rate of 10 °C/min, followed by an isothermal holding time of 30 min. Subsequently, the temperature was further increased to three target temperatures (1350 °C, 1400 °C, and 1450 °C), with another 30 min of isothermal holding at each temperature to ensure sufficient reaction. After completion of the holding process, the samples were rapidly cooled to room temperature in air using a fan, so as to preserve the phase assemblage at high temperatures.

### 2.3. Test Methods

#### 2.3.1. *f-CaO* Content in Clinkers

The *f-CaO* content of the quenched clinkers was quantitatively determined using the ethylene glycol method in accordance with Chinese Standard GB/T 176-2017 [15]. A mass of 0.5 g of the sample was mixed with 30 mL of glycerol–ethanol solution in a dried conical flask. One gram of strontium nitrate catalyst was introduced into the mixture. The samples were heated to gentle boiling with continuous stirring for 10 min to ensure complete alcoholysis of *f-CaO*. Afterwards, the mixture was immediately titrated with standard benzoic acid solution until the red color disappeared. The *f-CaO* content was calculated based on the total volume of benzoic acid solution consumed in the titration (Equation (1)). The above procedure was repeated three times, and the average value was taken as the test result.

(1) $$W_{f\text{-}\mathrm{CaO}}(\%)=\frac{T_{\mathrm{CaO}}\;\times \;V\;\times \;0.1}{m}$$

where *W_f-CaO_*: Mass fraction of *f-CaO*, %;
*T_CaO_*: Titration degree of benzoic acid–absolute ethanol standard titration solution to calcium oxide, mg/mL;
*V*: Volume of benzoic acid-absolute ethanol standard titration solution consumed during titration, mL;
*m*: Mass of the sample, g.

#### 2.3.2. X-Ray Diffraction

To complement the macroscopic burnability assessment, microstructural characterization was conducted. The phase identification and semi-quantitative analysis of the crystalline components in the clinker were performed using X-ray diffraction (XRD; Bruker D8 Advance, Karlsruhe, Germany) with Cu Kα radiation; the 2θ range from 5° to 70° was scanned at a rate of 0.2°/min. A 10% mass fraction of internal standard substance α-Al_2_O_3_ was incorporated into the clinker samples, and the K-value method was employed to calculate the mineral contents in the clinkers [16].

#### 2.3.3. Bogue Calculation for Mineral Content Determination

The XRF analysis from melt-fused beads was used to determine the chemical composition of clinkers. The loss on ignition (LOI) was determined by difference in weight; 1.5 g of sample material was placed in a preheated corundum crucible and heated to 1000 °C for 1 h. The mineralogical composition of the clinkers (i.e., C_3_S, C_2_S, C_3_A, and C_4_AF contents) was calculated from the chemical composition using the standard Bogue formulas [17]. The applicability of the Bogue formulas is limited to comparisons among cements with identical raw meal compositions. The Bogue calculation is an estimation based on an empirical formula derived from oxide contents and does not represent a direct measurement. In the present study, it is employed primarily for comparative trend analysis rather than as a basis for absolute values.

*C*_3_*S* = 4.07 × *C* − 7.6 × *S* − 6.72 × *A* − 1.43 × *F*

*C*_2_*S* = 2.87 × *S* − 0.754 × *C*_3_*S*

*C*_3_*A* = 2.65 × *A* − 1.69 × *F*

*C*_4_*AF* = 3.04 × *F*

where

- *C*: Content of CaO in clinker, %;
- *S*: Content of SiO_2_ in clinker, %;
- *A*: Content of Al_2_O_3_ in clinker, %;
- *F*: Content of Fe_2_O_3_ in clinker, %.

#### 2.3.4. Petrographic Characteristic

Furthermore, a microscope (ZEISS Axio Scope A1, Jena, Germany) was employed for microstructural observation. The quenched clinker nodules were mounted in epoxy resin, polished to a 1 μm finish, and etched with a solution of nitric acid and ethanol to reveal the crystal boundaries. To characterize the microstructure, the morphology, size, and distribution of alite (C_3_S), belite (C_2_S), and interstitial phases were examined. Reflected light microscopy was employed for imaging.

#### 2.3.5. BSE Imaging and SEM-EDX

Initially, the bulk clinker specimens were precisely sectioned into appropriate dimensions using a high-precision diamond wire saw. The specimens underwent sequential grinding with silicon carbide abrasive papers, progressing from coarse to fine grits (up to P2500). Following this, a final polishing step was performed utilizing an argon ion broad-beam milling system, which effectively removed the subsurface damage layer and circumvented mechanical strain induction. The samples were ultrasonically cleaned in anhydrous ethanol and dried under a nitrogen gas stream. A thin, uniform layer of conductive carbon was deposited onto the surfaces via sputter coating. This step was crucial to prevent charging effects during SEM-EDX analysis, thereby yielding pristine crystalline surfaces suitable for high-quality signal acquisition.

The polished clinker samples were examined in a high-resolution dual-beam SEM (ZEISS-Sigma 300, Jena, Germany), equipped with a silicon drift EDX detector (XMAX 80, Oxford Instruments, High Wycombe, UK). SEM-EDX mapping was recorded at 12 kV acceleration voltage and a moderate beam current of 0.8 nA. Spectra were recorded using a step size of 0.3 µm and a dwell time of 0.3 ms. Drift correction was employed during the acquisition to maintain spectral fidelity.

## 3. Results and Discussion

### 3.1. Influence of Particle Size on f-CaO Content

#### 3.1.1. *R*_80μm_ of Calcareous Materials

**Figure 1:**
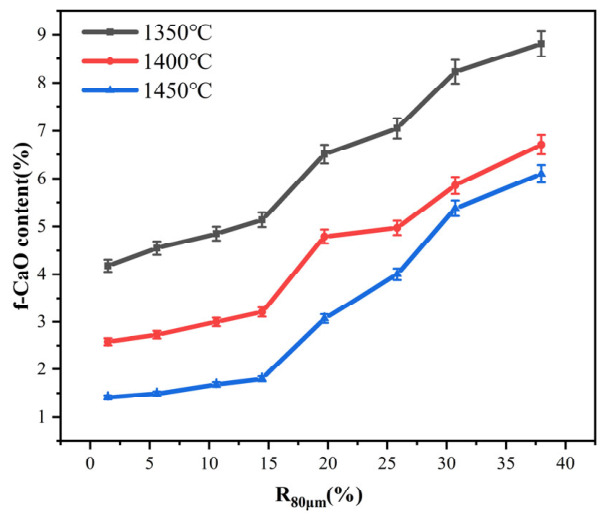
Effect of calcareous material fineness and temperature on *f-CaO* content.

As the most direct and reliable indicator of clinker burnability, the *f-CaO* content shows a strong, systematic dependence on calcination temperature and raw meal particle size. This relationship is clearly demonstrated in Figure 1. Segmented regression analysis reveals a distinct two-phase threshold effect of *R*_80μm_ on *f-CaO* content. In the fine-particle range (*R*_80μm_ < 15%), the increase in *f-CaO* content is relatively gradual, with the curve showing notable flattening at 1450 °C. This indicates that fine particles, due to their high specific surface area and reactivity, promote the consumption of *f-CaO*. As a result, temperature demonstrates a significantly stronger influence on *f-CaO* than particle size within this range. When *R*_80μm_ exceeds 15%, the *f-CaO* content shows a pronounced upward trend at 1350 °C, 1400 °C, and 1450 °C. This steep rise highlights the dominant role of coarse particles. Their lower reactivity results in a higher content of unreacted CaO. For any given sample, the *f-CaO* content demonstrates a monotonic decrease with increasing temperature. This behavior aligns with the principles of solid-phase reaction kinetics. At elevated temperatures, thermal energy exponentially enhances the diffusion rates of Ca^2+^ and Si^4+^ ions within the liquid phase and the newly formed silicate crystals. Consequently, the conversion of residual lime into tricalcium silicate (C_3_S, alite) is accelerated [18].

Figure 1 demonstrates that the calcareous constituents exhibit a critical fineness threshold, quantified by an *R*_80μm_ of approximately 15%. Below this threshold, the system exhibits excellent burnability even at 1400 °C. Beyond this point, a nonlinear, sharp deterioration in burnability is observed. This threshold likely marks a microstructural transition. At this point, the surface area of CaO particles becomes insufficient to sustain adequate diffusion fluxes for complete reaction within the given time frame. As a result, unreacted lime becomes encapsulated [19].

#### 3.1.2. R_200 μm_ of Calcareous Materials

**Figure 2:**
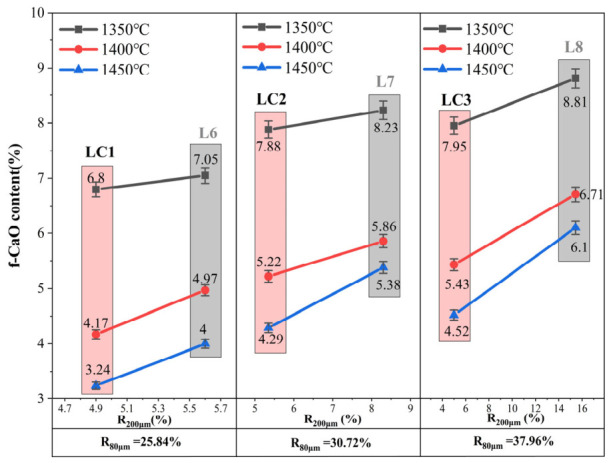
Influence of 200 μm coarse particles on *f-CaO* content.

The results in Figure 2 indicate that under similar *R*_80μm_ conditions, the *f-CaO* content increases significantly with a higher proportion of coarse particles (*R*_200μm_). For instance, samples L8 and LC3, both with *R*_80μm_ around 37.96%, demonstrate this trend. When R_200 μm_ rises from 5.0% to 15.44%, the *f-CaO* content at 1450 °C increases from 4.52% to 6.1%. Compared with particles larger than 80 μm, those exceeding 200 μm exhibit a stronger influence on *f-CaO* content at the same temperature, as shown by a comparison between Figure 1 and Figure 2. This difference underscores the critical role of particles >200 μm as a “diffusion barrier” during clinker formation [20]. Furthermore, the slope of *f-CaO* increase with *R*_200μm_ is notably steeper at 1450 °C than at 1350 °C and 1400 °C. Higher temperatures enhance reaction kinetics and promote microstructural evolution. These factors accelerate the surface reactivity of CaO in coarse particles, leading to a steeper rise in *f-CaO* content within the same size range.

During clinker formation, the generation of C_3_S is highly dependent on a liquid-phase-mediated “dissolution–precipitation” mechanism, wherein C_2_S and CaO initially dissolve into the high-temperature melt, and C_3_S nuclei subsequently precipitate under conditions far from thermodynamic equilibrium. However, the presence of coarse calcareous particles disrupts this chemical equilibrium. According to Wagner’s diffusion theory, the rate of a solid-state reaction is governed by the migration rate of the slowest-diffusing ion through the product layer, and the time required for complete diffusion is proportional to the square of the diffusion distance [21]. When calcareous particles larger than 200 µm are present in the raw meal, the characteristic diffusion distance increases exponentially. As the external reaction proceeds, the early-formed dense C_2_S rim acts as a physical barrier, further inhibiting the decomposition of CaCO_3_ and the dissolution of CaO, thereby creating a “self-locking effect” that encapsulates highly reactive Ca within the core of the coarse particles. These coarse grains are not readily wetted or engulfed by the high-temperature liquid phase and tend to persist as isolated, slow-reacting “unreacted cores.” Ultimately, upon cooling in the clinker cooling zone, these coarse unreacted cores accumulate and manifest as *f-CaO* [22].

Even more notably, coarse particles introduce microstructural heterogeneity. This leads to insufficient local liquid phase content and increased melt viscosity during sintering. Consequently, ion transport and alite (C_3_S) formation are further suppressed. Such structural defects cannot be fully compensated for merely by increasing the sintering temperature and may instead result in energy waste and operational instability [23].

### 3.2. Mineral Compositions of Clinkers

**Figure 3:**
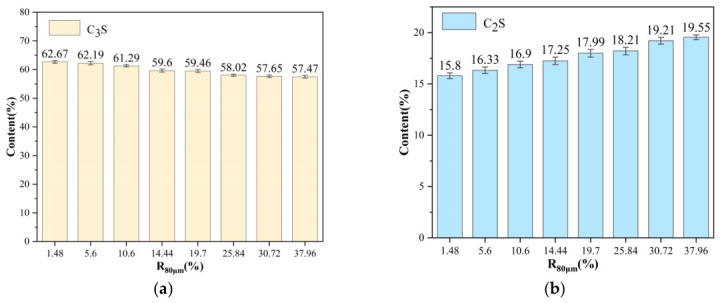

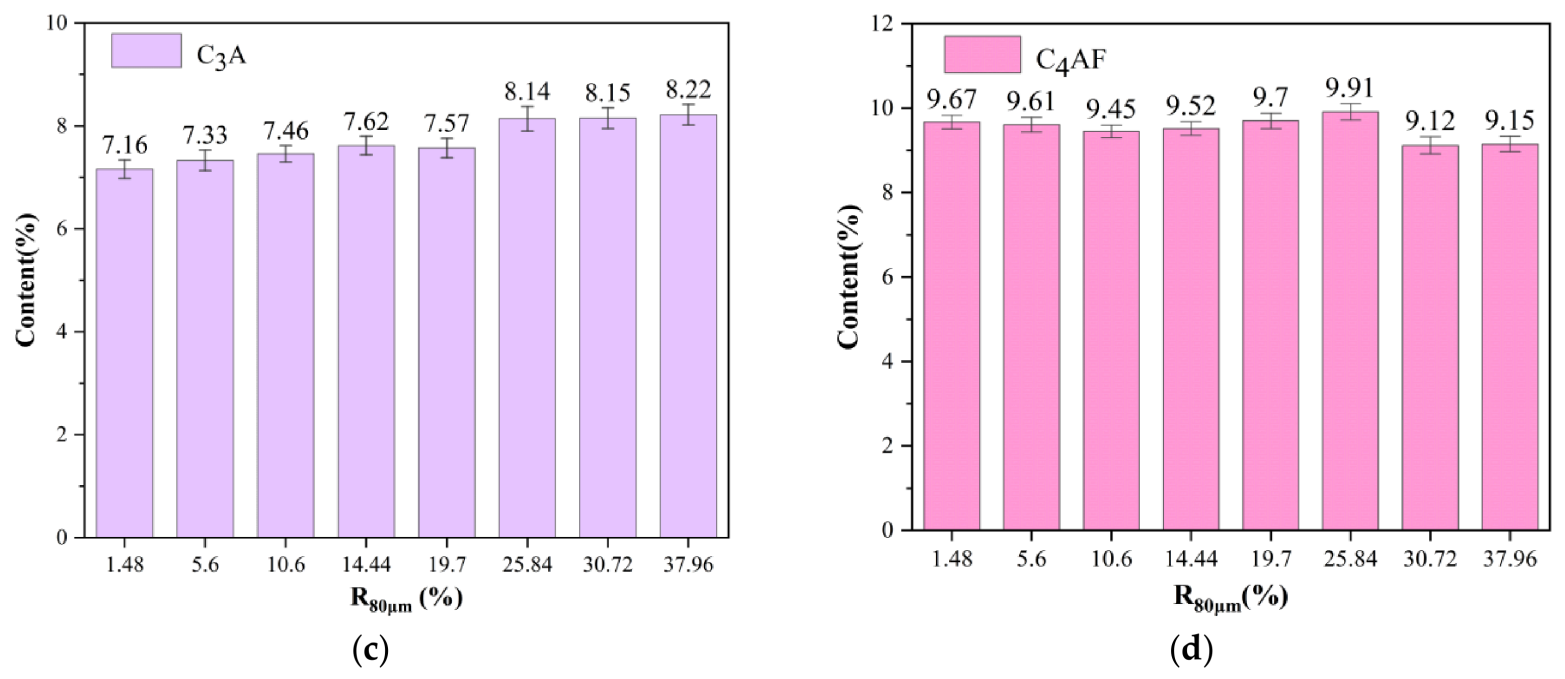
Effect of the fineness of calcareous material on cement clinker minerals: (**a**) C_3_S content; (**b**) C_2_S content; (**c**) C_3_A content; (**d**) C_4_AF content.

The calculated mineralogical composition of the clinkers, derived via the Bogue formulas [24,25], provides a crucial mechanistic link between the raw meal’s physical properties (fineness) and its macroscopic burnability (*f-CaO*). The evolution of the four primary clinker phases (C_3_S (alite), C_2_S (belite), C_3_A, and C_4_AF) not only confirms the *f-CaO* trends but also unveils the underlying reaction pathways and bottlenecks induced by particle size variation. Representative mineralogical compositions (Bogue-calculated, wt. %) of clinkers fired at 1450 °C can be seen in Figure 3.

As illustrated in Figure 3a, a strong negative correlation exists between the final C_3_S content and the calcareous component’s *R*_80μm_. The finest calcareous meal (L1) yielded a high C_3_S content of approximately 62.67%, which progressively decreased to about 57.47% for the coarsest meal (L8) at 1450 °C. This decline arises directly from an incomplete reaction. Coarse CaO particles fail to fully integrate into the liquid phase and react with C_2_S within the given thermal budget. This behavior is consistent with the dissolution–diffusion–precipitation model of alite formation [26]. Conversely, the belite (C_2_S) content exhibits a complementary increase with coarser calcareous fineness (Figure 3b). This accumulation of the intermediate phase C_2_S is a clear mineralogical marker of arrested clinkerization kinetics. The system remains trapped in a metastable state where the formation of C_2_S from CaO and SiO_2_ has occurred, but the subsequent reaction C_2_S + CaO → C_3_S is hindered by the limited availability of reactive, finely divided lime [27].

Figure 3c,d show that as the percentage of *R*_80μm_ increases from 1.48% to 37.96%, the C_3_A content exhibits an overall upward trend, with values rising from approximately 7.16% to nearly 8.22%. In contrast, C_4_AF content fluctuates between 9% and 10%, with no significant upward or downward trend. This indicates that changes in the sieve residue have a relatively minor impact on the formation of this mineral phase [28].

The mineralogical evolution underscores a fundamental shift in reaction mechanism governed by particle size. The fine calcareous meals promote a direct and efficient pathway to high-alite clinker, while coarse calcareous meals result in a stalled reaction sequence characterized by belite accumulation and unreacted lime. This provides a profound theoretical basis for the observed macro-scale burnability differences.

**Figure 4:**
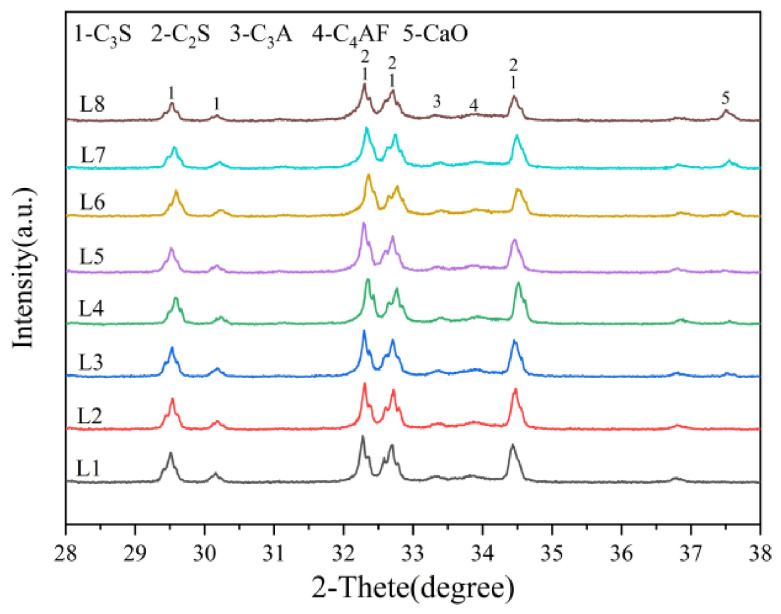
XRD patterns of clinker minerals with different fineness of calcareous material at 1450 °C.

The XRD patterns (presented in Figure 4) provide a definitive crystallographic fingerprint of the reaction completeness. Clinkers derived from fine calcareous meals (e.g., L1) exhibit XRD patterns characterized by sharp and intense diffraction peaks for alite (C_3_S), notably the strong reflections near 32.1° and 34.3° 2θ [29]. Concurrently, the peaks for belite (C_2_S) and *f-CaO* are markedly diminished or absent. This indicates a high degree of crystallinity and complete consumption of reactants.

In stark contrast, the XRD patterns of clinkers from coarse meals (e.g., L8) tell a different story: belite peaks (e.g., around 31.2° and 32.8° 2θ) are significantly more intense, and a discernible CaO peak is present near 37.5° 2θ [30]. Furthermore, the alite peaks are broader and less intense, indicative of smaller crystal size and/or microstrain within the crystals, resulting from hindered growth in a matrix with persistent, unreacted components [31]. This provides direct crystallographic evidence of the reaction arrest predicted by the Bogue calculations and the kinetic model. The evolution of the relative intensity ratio of the major alite to belite peaks can serve as a semi-quantitative microstructural index of burnability, perfectly aligning with the *f-CaO* trends.

**Figure 5:**
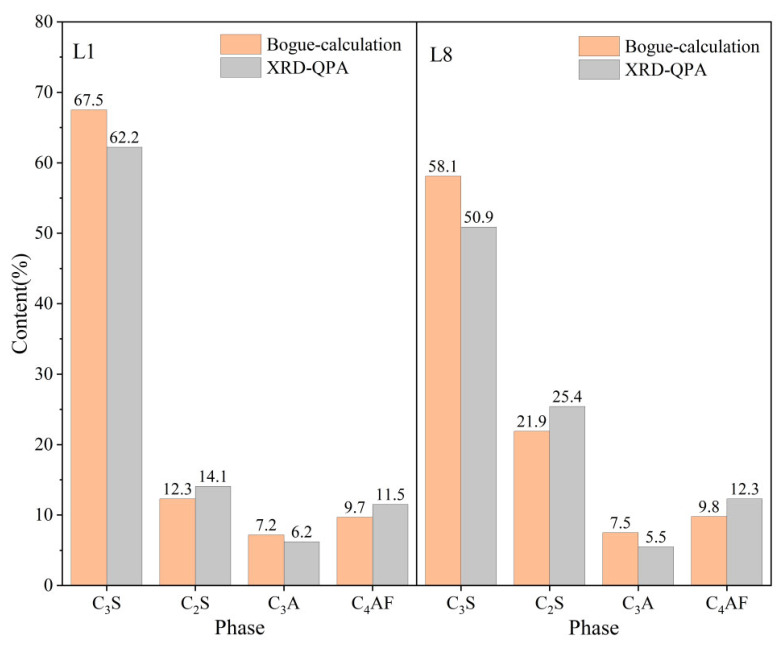
Differences in clinker mineral content under Bogue calculations and XRD-QPA conditions.

The differences in clinker mineral content under Bogue calculations and XRD-QPA conditions are shown in Figure 5. As shown in Figure 5, systematic discrepancies exist between the quantitative phase analysis (XRD-QPA) results obtained through Rietveld refinement and the conventional Bogue calculation values based on chemical oxides. The Bogue calculation yields a significantly higher C_3_S content than the XRD-QPA results. This is attributed to the fact that the Bogue formulas assume the formation of pure-phase minerals under ideal stoichiometry from all calcium oxide, without accounting for solid–solution effects [32]. In practice, significant amounts of Al_2_O_3_, Fe_2_O_3_, and minor impurities (e.g., MgO and SO_3_) incorporate into the C_3_S crystal lattice. This results in the formation of non-stoichiometric alite solid solutions [30,33]. XRD-QPA, via Rietveld refinement, directly refines the actual crystal structures (including solid solutions), thereby possibly providing a more accurate representation of the alite phase content [34].

The XRD-QPA results for L1 and L8 indicate that increasing the proportion of coarse limestone particles leads to a decrease in the C_3_S content and a notable increase in the C_2_S content within the clinker. This is due to incomplete reactions caused by the coarse limestone particles, resulting in SiO_2_ persisting in amorphous or under-reacted belite forms, which can be partially identified by XRD-QPA. Furthermore, with coarser limestone particles, not only is the total amount of C_3_S formed reduced, but the increased non-equilibrium nature of the system may also lead to more complex solid–solution compositions. This further widens the gap between theoretical calculations and actual measured values.

### 3.3. Analysis of Microstructure and Compositional Distribution

**Figure 6:**
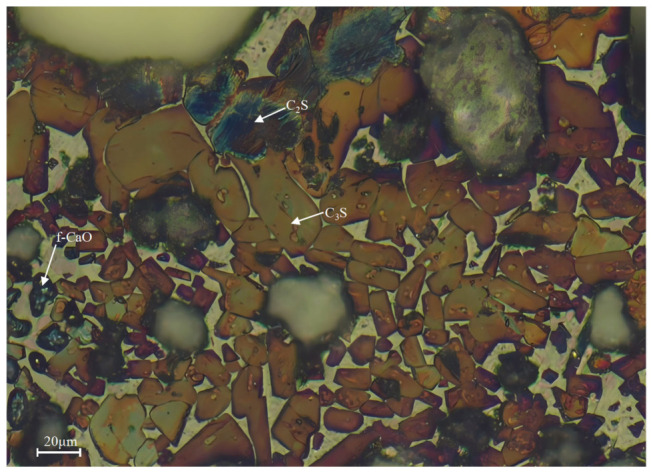
Petrography of cement clinker obtained at 1450 °C (L1).

**Figure 7:**
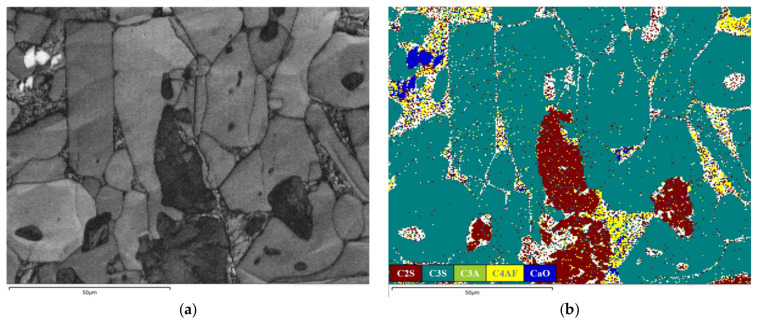

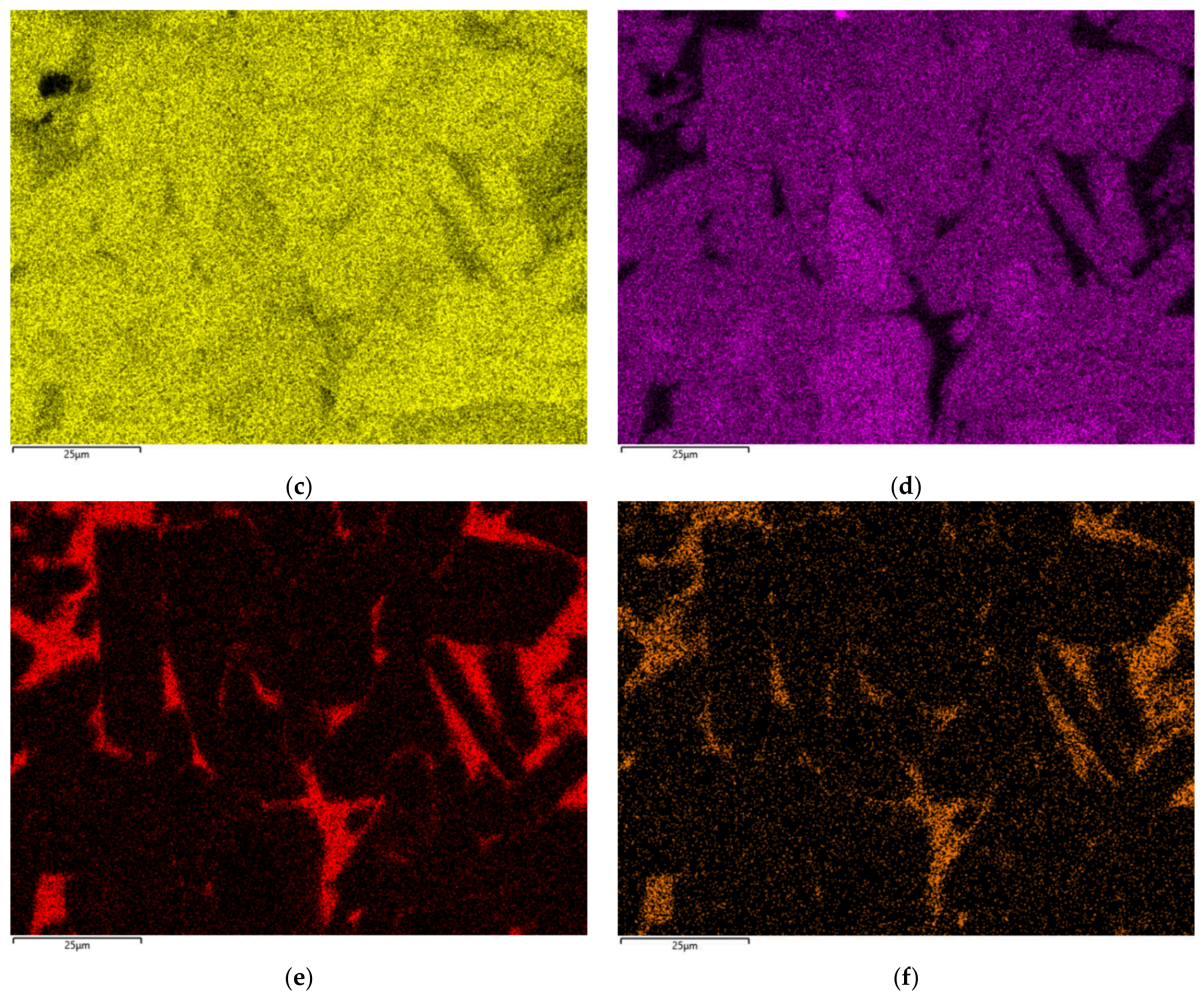
BSE image and SEM-EDX mapping data of L1: (**a**) BSE; (**b**) Phase map; (**c**) Ca; (**d**) Si; (**e**) Al; (**f**) Fe.

The clinker microstructure derived from fine limestone raw meals (L1) exhibits a highly uniform and well-developed character (Figure 6 and Figure 7). Alite (C_3_S), as the most critical hydraulic mineral in clinker, is highly representative in terms of its crystallographic morphology and spatial distribution. In the micrographs of sample L1, alite crystals predominantly appear as well-defined hexagonal plates and prismatic forms. Based on petrographic and SEM-EDX analyses, the dimensions of at least 200 alite crystals were quantified from BSE images using ImageJ software (version 1.54s). The statistical analysis yields an average crystal size of 31.45 μm, with the distribution predominantly falling within the range of 20–40 μm. This indicates a synchronized and controlled nucleation and growth process within an environment characterized by a uniform chemical potential field [35,36]. The development of such favorable crystal habits is attributed to sufficient ionic supply and gentle concentration gradients. These conditions provide abundant Ca^2+^ and silicate ions, enabling the crystals to grow into thermodynamically stable morphologies according to their intrinsic crystallization tendencies [37]. This presents a direct contrast to the anhedral, finer, and fragmented alite crystals observed in the coarse-meal sample.

The distribution and morphology of belite (C_2_S) further corroborate the homogeneity of the system. Belite crystals generally appear as fine, rounded, elliptical or circular grains, with sizes smaller than those of alite. They are uniformly dispersed within the interstitial matrix composed of the aluminate (C_3_A) and ferrite (C_4_AF) phases, rather than forming clusters. This dispersed state suggests that the silica component was fully and uniformly diffused during the formation of the liquid phase, with no localized SiO_2_-rich zones [38]. Consequently, this prevented the abnormal, excessive accumulation of belite as an intermediate product of incomplete reaction.

The interstitial phases, primarily tricalcium aluminate (C_3_A) and tetracalcium aluminoferrite (C_4_AF), formed during the final cooling stage, constitute a continuous and well-enveloping matrix. The formation of this continuous matrix indicates a uniform raw meal chemical composition and an appropriate firing regime [39]. C_3_A displays a diffuse distribution pattern, manifesting as fine punctate or small clustered forms that are relatively evenly dispersed throughout the matrix. In contrast, C_4_AF predominantly occurs in aggregated forms, giving rise to several larger, denser agglomerates or continuous regions, thereby exhibiting a markedly non-uniform distribution. Furthermore, owing to the thoroughness of the reaction process, particles are tightly interconnected through extensive solid-phase reactions and liquid-phase sintering, resulting in only a minimal presence of unfilled pores.

In summary, the microstructure of sample L1 embodies the characteristics of a nearly ideal reaction system dominated by sufficient diffusion kinetics under near-equilibrium conditions with ample ionic supply. Analysis of the clinker microstructure reveals that optimizing raw meal fineness increases the reactant contact area. This reduces diffusion resistance and promotes a more uniform melt distribution. These improvements thereby provide a basis for guiding the microstructure toward higher performance.

**Figure 8:**
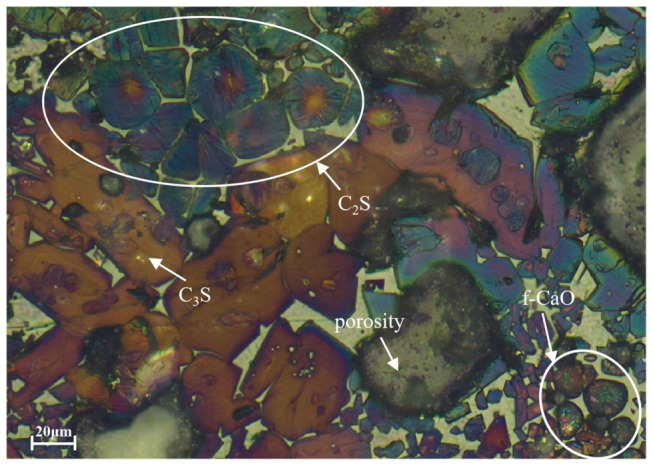
Petrography of cement clinker obtained at 1450 °C (L8).

**Figure 9:**
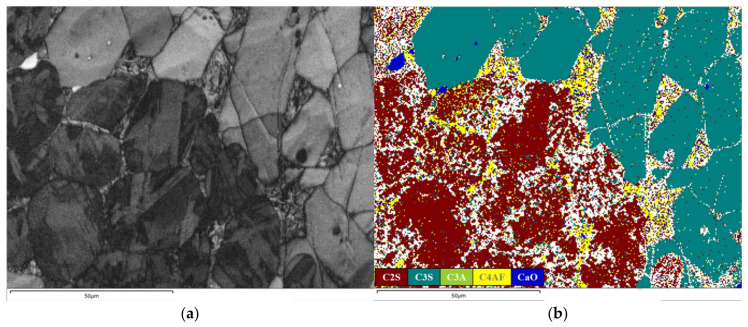

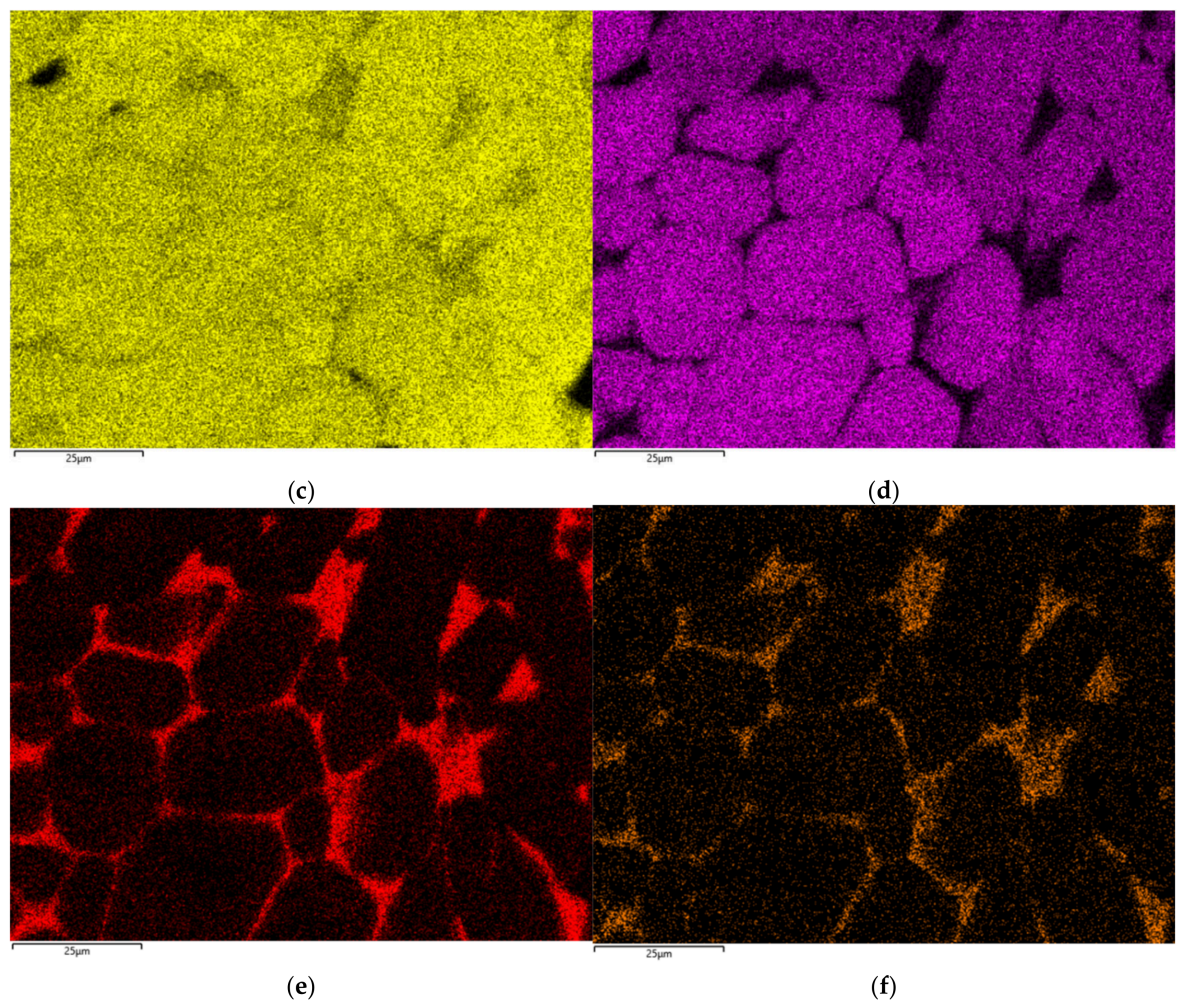
BSE image and SEM-EDX mapping data of L8: (**a**) BSE; (**b**) Phase map; (**c**) Ca; (**d**) Si; (**e**) Al; (**f**) Fe.

The clinker produced from coarse limestone raw meals (sample L8) exhibits a markedly heterogeneous and incompletely reacted microstructure (Figure 8 and Figure 9). On the micro-scale, coarse belite crystal agglomerates are prevalent, with a statistical mean grain diameter of 22.07 μm and a size distribution predominantly in the range of 18–30 μm. They exhibit distinct aggregation zones. These aggregates signify regions where silica is sufficiently available, but the diffusive flux of reactive lime (CaO) is severely restricted [40]. During high-temperature firing, the CaO derived from the decomposition of coarse limestone particles fails to diffuse in a timely manner into silica-rich zones, resulting in a persistently low local Ca/Si ratio. There is also a clear boundary in the distribution of Si elements (Figure 9d). This condition promotes the substantial formation and anomalous growth of belite as an intermediate phase [41], providing direct evidence for the dominance of the diffusion-controlled mechanism in the solid-state reactions.

The microstructure distinctly reveals residual, unreacted coarse CaO particles, often surrounded by a rapidly crystallized belite rim, forming a characteristic core–shell structure. This morphology represents a spatial manifestation of diffusion barriers. The lime core becomes physically isolated by the initially formed belite layer. This isolation prevents further contact with the external aluminosilicate melt, effectively arresting the reaction at an intermediate stage. Notably, this core–shell configuration not only reduces the conversion efficiency of lime but may also pose a potential risk for long-term volume instability during clinker hydration [42].

Adjacent to these coarse residues, the size and abundance of alite (C_3_S) crystals are significantly diminished, creating a conspicuous “alite-depleted zone.” The few alite crystals that do form in these regions are predominantly anhedral with poorly developed crystal faces. This reflects the strong constraints imposed on crystal growth by local compositional fluctuations and competitive crystallization processes within a chemically inhomogeneous environment with a discontinuous diffusion field [43]. The nucleation and growth of alite depend critically on two conditions: chemical homogeneity of the surrounding melt and an ample supply of calcium. However, the compositional heterogeneities introduced by coarse raw meals disrupt precisely these conditions.

On a more macroscopic scale (Figure 9), these manifestations of incomplete reaction collectively lead to an overall increase in the material’s porosity. The failure of solid-state reactions to reach completion results in insufficient filling between particles, leaving behind numerous interparticle gaps and voids not filled by the liquid phase. This porous structure not only reduces the apparent density of the clinker but may also compromise its ultimate mechanical strength and durability.

These microstructural features are entirely consistent with the previously observed XRD phenomena. Specifically, the alite diffraction peaks were broadened, the belite peaks were relatively enhanced, and *f-CaO* persisted. They provide direct visual evidence at the microstructural level for the limitation of reaction kinetics [44].

In summary, the clinker produced by calcining coarse limestone raw meals exhibits a series of interrelated microstructural defects. These defects originate from obstructed reaction pathways dominated by diffusion control, resulting in uneven phase distribution, incomplete development of key crystalline phases, and elevated porosity. Research on the influence of calcium-bearing material particle size on the formation of clinker microstructure can provide a scientific basis for optimizing process control parameters in production and enhancing clinker performance.

### 3.4. Reaction Kinetics Model

#### 3.4.1. Model Establishment

A semi-empirical reaction kinetics model was developed to quantify the coupled effects of calcination temperature (T) and calcareous component fineness (*R*_80μm_) on the residual *f-CaO* content. This approach transcends empirical observations and provides a predictive tool for industrial optimization. The modeling approach is based on a key premise. The clinker calcining process, particularly the consumption of *f-CaO*, is governed by diffusion-controlled kinetics [45]. The rate of *f-CaO* consumption is proportional to the ionic diffusion flux. This flux is governed by two key factors: it increases exponentially with temperature and is inversely related to the diffusion path length, a parameter intrinsically linked to the raw meal particle size.

The relationship for the final *f-CaO* content after a fixed isothermal holding time is described by Equation (2):

(2) $$f\text{-}\mathrm{CaO}\;=\;A\cdot \exp\frac{E_{a}}{\mathrm{RT}}\cdot {\left(R_{80\;\mu m}\right)}^{n}$$

where

*A* is the pre-exponential factor, encompassing geometric and frequency factors.
*E_a_* (kJ·mol^−1^) is the apparent activation energy for the clinker calcining process. It is proposed to be a function of the particle size distribution, rather than a constant.
*R* is the universal gas constant (0.008314 kJ·mol^−1^·K^−1^).
*T* is the absolute sintering temperature (K).
*n* is the fineness factor that quantifies the sensitivity of the reaction rate to particle size.
*R*_80μm_ is the 80 μm sieve residue (%).

The most significant theoretical innovation of this model is the introduction of a size-dependent activation energy, E_a_. Traditional models often assume E_a_ is constant for a given chemical reaction [46]. However, clinkerization is a complex multi-phase process in which diffusion through a viscous melt and product layers is rate-limiting. For such processes, the energy barrier is profoundly influenced by microstructural features. Coarser particles increase the diffusion path length for ions and create microstructural heterogeneities, effectively raising the apparent energy barrier that must be overcome for the reaction to proceed to completion. The relationship was modeled as a linear function (Equation (3)):

(3) $$E_{a}=\;E_{a,\;0}\;+\;k\;\times \;R_{80\;\mu m}$$

where *E_a,_*_0_ is the intrinsic activation energy for a theoretically infinitely fine powder, and k (kJ·mol^−1^·%^−1^) is an incremental factor.

This linear assumption is consistent with the kinetic theory of diffusion-controlled reactions. In solid-state reactions, the apparent activation energy E_a_ can be approximated as the sum of the energy barrier to overcome chemical bonds (the intrinsic activation energy, *E_a,_*_0_) and the barrier for diffusion (ΔE). The latter is proportional to the square of the diffusion distance (according to the Einstein–Smoluchowski relation, diffusion time τ ∝ r^2^/D) [20]. The *R*_80μm_ is directly related to the characteristic particle size (*r*). Therefore, the increase of *E_a_* with *R*_80μm_ can be interpreted as a linearized expression of the additional energy barrier due to increased diffusion path lengths. Future work could employ computer simulations (e.g., Monte Carlo or Phase-Field methods) to establish a more precise quantitative relationship between particle size and the diffusion barrier.

#### 3.4.2. Model Verification and Applicability Analysis

**Figure 10:**
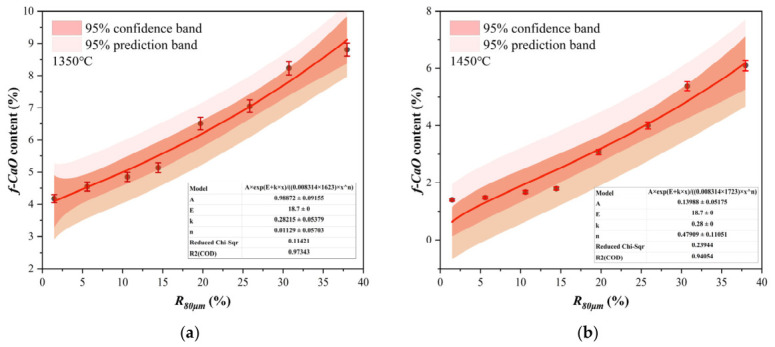
Fitting of model parameters for *f-CaO* under different temperatures: (**a**) 1350 °C, (**b**) 1450 °C.

Based on the comprehensive dataset of the *f-CaO*, nonlinear regression analysis was employed to fit the parameters of Equation (2). The relevant parameters are summarized in Table 6 and illustrated in Figure 10. The model exhibited excellent goodness of fit (*R*^2^ > 0.95), confirming its predictive capability. The fitted intrinsic activation energy (*E_a,_*_0_) is approximately 18.7 kJ·mol^−1^. This value is consistent with reported values for diffusion-controlled processes in silicate melts [47]. Crucially, the model quantitatively confirmed that the incremental factor *k* is positive, indicating that the apparent activation energy *E_a_* increases with rising *R*_80μm_ This parameter holds significant practical implications. Based on the fitted value *k* = 0.28 kJ·mol^−1^·%^−1^, a 1% reduction in the *R*_80μm_ of raw meal corresponds to a decrease of approximately 0.28 kJ·mol^−1^ in the apparent activation energy required for clinker sintering. For instance, the fine raw meal (L1, *R*_80μm_ = 1.48%) exhibited an apparent activation energy of approximately 19.11 kJ·mol^−1^, whereas that of the coarse raw meal (L8, *R*_80μm_ = 37.96%) increased to 29.33 kJ·mol^−1^.

This relationship can be further interpreted as achieving a lower *f-CaO* content under the same sintering regime, or realizing a reduction in theoretical sintering temperature (ΔT) while maintaining a target *f-CaO* content. Calculations were performed based on the Arrhenius equation and production practice. For this system, reducing *R*_80μm_ from 20% to 10% is approximately equivalent to lowering the sintering temperature by 100–200 °C while maintaining the same degree of reaction completion. This model provides a theoretical framework for quantifying the “mechanical-thermal energy trade-off.” It offers a quantitative scientific basis for life cycle assessment (LCA) and cost–benefit analysis in cement plants, facilitating the identification of the optimal solution that minimizes the total energy consumption of grinding and sintering processes [48].

Nonlinear regression analysis revealed that the n value increases significantly with sintering temperature, ranging from 0.01 at 1350 °C to 0.48 at 1450 °C. This variation indicates a substantial transition in the underlying mechanism through which fineness influences the process. At lower temperatures (1350 °C), interfacial chemical reactions coexist with solid-state diffusion. Under such conditions, the effect of fineness is primarily exerted through altering the diffusion energy barrier (i.e., activation energy). At higher temperatures (1450 °C), the system enters a purely diffusion-controlled regime. In this case, fineness affects the diffusion coefficient via activation energy. Moreover, it imposes a significant nonlinear impact on the reaction rate by modifying the geometric conditions of diffusion, including interfacial area, product layer structure, and liquid phase distribution.

**Figure 11:**
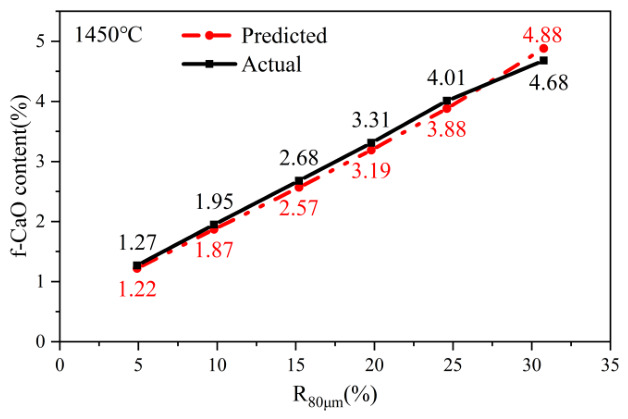
Comparison between model predictions and measured values.

Six validation tests were conducted using samples from Taishan Zhonglian, as shown in Figure 11. When the fineness of the calcareous component reached *R*_80μm_ = 15.2%, the *f-CaO* content was 2.68%. Under this threshold, the kinetic model predicted an *f-CaO* value of 2.57%, showing strong consistency with the experimental measurement within the investigated temperature range. The relative error was 4.3%, confirming the reliability of the model in describing the coupled effects of temperature and particle size. These validation results demonstrate that the proposed model can effectively capture the transition in the fineness-dependent reaction mechanism, providing a reliable tool for optimizing raw meal fineness.

This model is applicable under separate grinding conditions, with sintering temperatures ranging from 1350 °C to 1450 °C and *R*_80μm_ of calcareous materials between 0% and 40%. It provides a quantitative framework for process optimization. For any given raw meal fineness, the sintering temperature required to achieve a target *f-CaO* content can be predicted by adjusting the key model parameters (*A, E_a_, k, n*). Conversely, the model can determine the grinding fineness necessary to maintain product quality while reducing kiln temperature. The concept of variable activation energy (E_a_) offers a profound theoretical insight: investing energy in finer grinding (mechanical energy) effectively lowers the subsequent thermal energy barrier required for clinker formation. This provides a rigorous scientific basis for evaluating the trade-off between grinding energy consumption and sintering energy consumption. This trade-off is a critical consideration in lifecycle assessment and carbon footprint reduction in cement manufacturing [49].

The laboratory pressing and calcination method employed in this study (utilizing a static muffle furnace with fixed heating rates and holding times) differs intrinsically from the dynamic conditions prevailing in industrial rotary kilns, which are characterized by material tumbling, complex temperature gradients, and continuous bed movement. Consequently, direct extrapolation of the present model to industrial-scale production necessitates careful correction and adaptation based on specific kiln configurations and operational parameters. Future studies are recommended to externally validate the model using a broader range of independent samples, including variations in LSF, SM, AM, and raw material provenance, to rigorously assess its generalizability and robustness.

In addition to the foregoing discussion, two limitations of the present study should be explicitly acknowledged. First, the characterization of particle size relies on *R*_80μm_ sieve residue. Although this parameter is of practical relevance to the cement industry, it neglects the potential influence of particle size on sintering behavior. Second, this work is confined to the clinker burning stage and does not extend to the compressive strength development, hydration kinetics, or long-term durability of the corresponding cement. These end-use properties are essential for evaluating the practical engineering benefits of particle size control. Future research will address these limitations by incorporating complete particle size distribution analysis and cement performance testing.

## 4. Conclusions

(1) The fineness of calcium components has been identified as the main factor determining clinker burnability, with a critical threshold (*R*_80μm_ = 15%). When the particle size falls below this threshold, the raw meal exhibits excellent burnability, with temperature playing the dominant role in influencing *f-CaO*. In the presence of equivalent levels of *f-CaO*, the optimal temperature for the reaction can be reduced to 1400 °C. When the particle size exceeds the critical threshold, diffusion limitations intensify, and coarse particles become the dominant factor. The lower reactivity of the samples results in a higher content of unreacted CaO, leading to a significant increase in the *f-CaO* content in a nonlinear manner.
(2) It is evident that as the proportion of 200 μm coarse particles in the sample increases, there is a concomitant rise in the *f-CaO* content. Furthermore, 200 μm particles have been demonstrated to exert a greater influence on *f-CaO* than 80 μm particles. The presence of coarse particles has been shown to significantly prolong the solid-phase diffusion pathways for ions such as Ca^2+^ and SiO_4_^4−^, thereby causing the time required for complete reaction to increase exponentially. This hinders the liquid-phase-mediated conversion of C_2_S and CaO into C_3_S. Concurrently, coarse particles are not fully wetted or encapsulated by the high-temperature melt. This results in the persistence of isolated, unreacted cores that remain inert during the reaction. The process leads to the localized enrichment of *f-CaO*.
(3) When the *R*_80μm_ exceeds the critical threshold, significant changes occur in both the phase composition and the microstructure of the clinker. First, Bogue calculations and QXRD analysis reveal a decrease in alite C_3_S content, accompanied by a gradual enrichment of belite C_2_S and *f-CaO*. The X-ray diffraction pattern transitions from exhibiting sharp and intense C_3_S peaks to showing enhanced C_2_S peaks along with clearly identifiable *f-CaO* reflections. Second, in terms of microstructure, petrographic and BSE-EDX analyses indicate that the clinker evolves from a homogeneous and dense ideal structure rich in C_3_S to a heterogeneous one characterized by the formation of CaO cores, the presence of C_2_S agglomerates, and the development of poorly formed C_3_S crystals.
(4) A semi-empirical reaction kinetic model $f\text{-}\mathrm{CaO}\;=\;A\cdot \exp\frac{E_{a}}{\mathrm{RT}}\cdot {\left(R_{80\;\mu m}\right)}^{n}$ incorporating fineness-dependent activation energy was developed based on diffusion-controlled kinetics. This approach overcomes the conventional assumption of constant activation energy by establishing a constitutive relationship expressed as $E_{a}=E_{a,\;0}+k\times R_{80\;\mu m}$. The model exhibited excellent goodness of fit (*R*^2^ > 0.95), with an intrinsic activation energy *E_a,_*_0_ of 18.7 kJ·mol^−1^ and an incremental factor k of 0.28 kJ·mol^−1^·%^−1^, quantitatively revealing the physical mechanism by which coarse particles extend diffusion pathways and increase energy barriers. Validation experiments yielded a relative error of 4.3%, confirming the reliability of the model. For application, the model may serve as a reference for the synergistic optimization of grinding power consumption and sintering energy consumption.

## Figures and Tables

**Table 1:** Chemical composition of raw materials (%).

Samples	L.O.I	SiO_2_	Al_2_O_3_	Fe_2_O_3_	CaO	MgO	K_2_O	Na_2_O	SO_3_	Cl-
Limestone	42.06	3.14	1.02	0.68	49.36	3.18	0.35	0.04	0.095	0.023
Sandstone	0.73	95.18	1.43	1.37	0.04	0.35	0.36	0.04	0.020	0.007
Fly ash	6.38	51.20	26.00	7.36	5.08	1.32	1.02	0.44	0.560	0.024
Iron-soil sludge	1.42	60.11	4.46	23.84	4.00	3.89	0.58	0.52	0.910	0.010
Coal ash	0.00	53.40	32.03	4.66	2.06	1.48	0.92	0.66	2.150	0.002

**Table 2:** Raw meal proportions (%).

Item	Limestone	Sandstone	Fly Ash	Steel Slag	Coal Ash
Raw meal	83.17	5.50	6.44	3.82	1.07

**Table 3:** Size distribution of the grinding media in the ball mill.

Diameter (mm)	36.5	30.2	25.4	19.1	15.9	Total
Number of balls	43	67	10	71	94	285

**Table 4:** Testing of calcareous materials with different fineness.

Item	L1	L2	L3	L4	L5	L6	L7	L8
*R*_80μm_, %	1.48	5.60	10.60	14.44	19.70	25.84	30.72	37.96
*R*_200μm_, %	0.04	0.16	0.88	1.68	3.55	5.60	7.80	15.44
Specific Surface Area (cm^2^/g)	9430	6610	5300	4960	4600	4330	3900	3430

**Table 5:** Test of controlled coarse calcareous particles.

Item	LC1	LC2	LC3
*R*_80μm_, %	25.84	30.28	37.62
*R*_200μm_, %	4.90	4.95	5.00
Specific Surface Area (cm^2^/g)	4830	4300	3860

**Table 6:** Fitted parameters for the kinetics model (Equation (2)).

Parameter	1350 °C	1450 °C	Description
*A*	0.99	0.14	Pre-exponential factor
*E*_*a,* 0_	18.7 kJ·mol^−1^	18.7 kJ·mol^−1^	Activation energy
*k*	0.28 kJ·mol^−1^·%^−1^	0.28 kJ·mol^−1^·%^−1^	Activation energy increment factor
*n*	0.01	0.48	Particle size exponent

## Data Availability

The original contributions presented in this study are included in the article. Further inquiries can be directed to the corresponding authors.
